# Adherence to a Mediterranean-style eating pattern and risk of diabetes in a U.S. prospective cohort study

**DOI:** 10.1038/s41387-020-0113-x

**Published:** 2020-03-20

**Authors:** Lauren E. O’Connor, Emily A. Hu, Lyn M. Steffen, Elizabeth Selvin, Casey M. Rebholz

**Affiliations:** 1 Department of Epidemiology, Johns Hopkins Bloomberg School of Public Health, Baltimore, MD USA; 2 Division of Cancer Prevention, National Cancer Institute, National Institutes of Health, Rockville, MD USA; 3 Division of Epidemiology and Community Health, University of Minnesota School of Public Health, Minneapolis, MN USA

**Keywords:** Epidemiology, Risk factors

## Abstract

**Background:**

A Mediterranean-style eating pattern is consistently associated with a decreased diabetes risk in Mediterranean and European populations. However, results in U.S. populations are inconsistent. The objective of this study was to assess whether a Mediterranean-style eating pattern would be associated with diabetes risk in a large, nationally representative U.S. cohort of black and white men and women.

**Methods:**

Participants from the Atherosclerosis Risk in Communities study prospective cohort without diabetes, cardiovascular disease, or cancer at baseline (visit 1, 1987–1989; *n* = 11,991) were included (mean age 54 years, 56% female, 75% white). Alternate Mediterranean Diet scores (aMed) were calculated using the mean dietary intake self-reported at visit 1 and visit 3 (1993–1995) or visit 1 only for participants censored before visit 3. Participants were followed from visit 1 through 31 December 2016 for incident diabetes. We used Cox regression models to characterize associations of aMed (quintiles as well as per 1-point higher) with incident diabetes adjusted for energy intake, age, sex, race and study center, and education (Model 1) for all participants then stratified by race and body mass index (BMI). Model 2 included potential mediating behavioral and clinical measures associated with diabetes. Results are presented as hazard ratios and 95% confidence intervals.

**Results:**

Over a median follow-up of 22 years, there were 4024 incident cases of diabetes. Higher aMed scores were associated with lower diabetes risk [Model 1: 0.83 (0.73–0.94) for Q5 vs Q1 (*p*-trend < 0.001) and 0.96 (0.95–0.98) for 1-point higher]. Associations were stronger for black vs white participants (interaction *p* < 0.001) and weaker for obese vs normal BMI (interaction *p* < 0.01). Associations were attenuated but statistically significant in Model 2.

**Conclusions:**

An eating pattern high in fruits, vegetables, whole grains, legumes, nuts, and fish, and moderate in alcohol was associated with a lower risk of diabetes in a community-based U.S. population.

## Introduction

A Mediterranean-style eating pattern (Mediterranean pattern) is one of the eating patterns recommended by the American Heart Association, American College of Cardiology, and the Dietary Guidelines for Americans to reduce chronic disease risk^1,2^. While there are cultural variations in the foods and beverages included in a Mediterranean pattern, overall it is largely plant-based, relatively high in olive oil and seafood, but low in dairy, red meat, and refined grains^1,3,4^. Observational and experimental studies suggest that higher adherence to a Mediterranean pattern is associated with improved cardiovascular disease risk factors^5–7^, reduced risk of cardiovascular events^8,9^, and reduced cardiovascular-related mortality^9,10^. The American Diabetes Association recommends adherence to a Mediterranean pattern, as one of several potential strategies, to prevent cardiovascular complications in individuals with diabetes. However, evidence regarding how Mediterranean pattern adherence can improve diabetes risk for U.S. populations is inconsistent^11^.

It is unclear if a Mediterranean pattern, independent of a Mediterranean lifestyle, can reduce diabetes risk in US adult populations^11^. Mediterranean and European populations tend to be less racially and ethnically heterogeneous, have lower body mass indexes (BMI), be less sedentary, have larger social networks, and place a greater emphasis on rest and sleep compared to the general U.S. population^12,13^. In Mediterranean and other European settings, adherence to a Mediterranean pattern is consistently associated with a reduced risk of diabetes^14–16^. Yet, research about Mediterranean pattern adherence and diabetes risk in U.S. populations is limited and inconsistent^11^. Research is needed to assess whether potential benefits of a Mediterranean pattern are translatable to heterogeneous populations which place less emphasis on ideologies and behaviors of Mediterranean-like cultures. Further, benefits may vary by race and BMI. Being of a minority race and having a BMI > 25 kg/m^2^ are among the top recognized risk factors for diabetes in the U.S. and eating habits differ within these groups^17^.

The purpose of this study was to assess associations between Mediterranean pattern scores and incident diabetes in a U.S. community-based population of adults. We also explored consistency of associations within racial and BMI subgroups^17^.

## Methods

### Study population

We used data collected from participants in the Atherosclerosis Risk in Communities (ARIC) study. The primary aim of the ARIC study was to investigate atherosclerosis etiology and clinical sequelae as well as to assess how cardiovascular disease risk factors differ by race, sex, place, and time^18^. Investigators originally recruited middle-aged adults (45–65 years, *n* = 15,792) from four U.S. communities (Washington County, Maryland; suburban Minneapolis, Minnesota; Jackson, Mississippi; and Forsyth County, North Carolina) and performed baseline assessments in 1987–89 (visit 1). Participants returned for follow-up visits in 1990–92 (visit 2), 1993–95 (visit 3), 1996–98 (visit 4), 2011–13 (visit 5), 2016–17 (visit 6), and 2018–18 (visit 7). Each participating institution received ethical approval from an institutional review board and all participants provided informed consent at each visit. This study has been registered with clinicaltrials.gov (NCT00005131).

The total sample size for this analysis was 11,991 participants (Supplementary Fig. S1) at visit 1. Participants were excluded for the following reasons: (1) if they had prevalent or history of cardiovascular disease, diabetes, or cancer (*n* = 3318); (2) implausible energy intake (<600 or >4200, and <500 or >3600 kcal for males and females, respectively) derived from food frequency questionnaire (FFQ) responses or ≥10 missing FFQ items (*n* = 270); (3) participants identified as Asian (*n* = 28) or Indian (*n* = 14) due to small sample size; (4) participants identified as black from Maryland (*n* = 25) and Minnesota (*n* = 19) due to inability to decipher the influence of geographic region versus race with small samples^19^; (5) missing baseline covariates (*n* = 20); (6) missing follow-up time; and (7) missing food items needed to calculate the Mediterranean pattern score (*n* = 63).

### Dietary intake assessment

A trained interviewer administered a 66-item semi-quantitative FFQ and alcohol consumption-related questions to participants at visits 1 and 3. The ARIC FFQ was adapted from a previously validated FFQ^20^ to ascertain more details pertaining to fish intake, cooking fats, and baked goods^19^. Participants reported average food intake frequency of a pre-specified portion size of various food items during the previous year. Nutrient estimates of assessed food and beverage items were obtained from linking to the United States Department of Agriculture’s Food Composition Databases^21^ at Harvard Medical School’s Channing Laboratory^20^. Repeated measurement of dietary and alcohol intakes from visit 1 and visit 3 were averaged for all analyses to increase precision. Visit 1 intake data were used if participants developed diabetes or were censored before visit 3 or if they did not attend visit 3.

### Mediterranean-style eating pattern scoring system

Alternate Mediterranean Diet (aMed) scores, previously developed to assess Mediterranean pattern adherence in U.S. populations^22^, were calculated using the FFQ data. The aMed scoring system assigns 1 point for self-reported consumption above the cohort’s sex-specific median for intakes of total vegetables, total fruit, whole grains, nuts, legumes, fish, and ratio of monounsaturated fatty acids to saturated fatty acids (MUFA:SFA), 1 point for self-reported consumption below the cohort’s sex-specific median intake for red and processed meat, and 1 point for self-reported alcohol intake between 5–15 g/day for females and 10–25 g/day for males. Scores range from 0 to 9 points; higher scores presume higher adherence to a Mediterranean pattern. Designation of ARIC FFQ items for the aMed scoring system is provided in Supplementary Table S1.

### Incident diabetes ascertainment

Incident diabetes cases were identified according to the following criteria: (1) self-reported physician diagnosis, (2) self-reported use of diabetes-related medication during previous 2 weeks, (3) measured fasting blood glucose concentration ≥126 mg/dL, or (4) measured non-fasting blood glucose concentration ≥200 mg/dL. Self-reported physician diagnosis or self-reported diabetes medication usage was ascertained at study visits and via annual follow-up phone calls between study visits and after visit 4. This definition of incident diabetes has high specificity^23^. Participants who did not develop diabetes were censored for death, loss to follow-up, or administratively censored on 31 December 2016.

### Covariates

Age, race, education level (less than high school, high school or GED/vocational equivalent, and more than high school), smoking (current, former, never), and family history of diabetes (including both maternal and paternal) were ascertained via questionnaires at visit 1. A race-center variable was derived to represent whites and blacks at each study center. Physical activity was measured via the Baecke questionnaire, which converts self-reported physical activity from sports, leisure, and work into a 1–5 point score^24,25^. Height and weight were measured via standard protocols and BMI was calculated as body weight in kilograms divided by height in meters squared. Seated blood pressure was measured in triplicates separated by resting for 5 minutes with a random-zero sphygmomanometer. The mean of the second and third measurements was used in the analysis. Hypertension status was determined using the 2003 National Heart, Lung, and Blood Institute guidelines^26^, defined as systolic blood pressure ≥140, diastolic blood pressure ≥90 mm Hg, or current use of anti-hypertensive medication. All biomarkers measurement methods have been previously described and all ARIC protocols are available at https://sites.cscc.unc.edu/aric/cohort-manuals.

### Statistical methods

We compared visit 1 participant characteristics and eating patterns according to aMed quintiles. We used Cox proportional hazards regression models to estimate hazard ratios (HR) and corresponding 95% confidence intervals (CI) for associations between aMed scores (according to quintiles and per 1-point higher) and incident diabetes. We tested for linear trends across quintiles by modeling quintiles as an ordinal variable. We tested for linear splines (a knot at 2 points was significant) to visually depict the shape of the relationship between aMed scores and risk of incident diabetes across the full range of aMed scores. In addition to analyzing overall aMed scores, associations between individual aMed components and incident diabetes were assessed. Follow-up time in days from study visit 1 was used as the time metric. The proportional hazard assumption was assessed via log-log plots and Schoenfeld’s residual tests. Stata version 15 statistical software was used for all analyses (StataCorp, College Station, Texas, USA).

Three covariate structures were used in the Cox regression analyses. Model 1 was adjusted for energy intake and demographic variables of age, sex, race-center, and education level. Model 2 included all variables in Model 1 plus behavioral variables associated with risk of developing diabetes, including smoking status and physical activity. Model 3 included all variables in Model 2 plus potential clinical mediators of diabetes including fasting glucose (mg/dL), hypertension status (yes/no), low-density lipoprotein cholesterol (mg/dL), BMI category (normal, overweight, or obese), and family history of diabetes (yes/no) to test for a potential direct association between aMed scores and incident diabetes.

Race^27^ and baseline BMI^28^ were chosen a priori as potential effect modifiers. Sex and education level effect modifiers were assessed post hoc. Likelihood ratio tests were used to assess interaction by categorical BMI (excluding underweight participants, *n* = 117), race (with adjustment for center rather than race-center), educational level, and sex on associations between continuous aMed scores and incident diabetes as well as quintiles of aMed and incident diabetes. If the *p*-value for interactions were significant (*p* < 0.05) using Model 1, we then conducted stratified analyses with Models 1, 2, and 3.

## Results

Participants in higher aMed score quintiles were qualitatively more likely to have a higher education, have a higher physical activity level, were less likely to be current smokers and less likely to be obese compared to participants in lower aMed score quintiles (Table 1). Age, sex, race, fasting glucose, hypertension status, and LDL cholesterol were similar across quintiles.

**Table 1:** Baseline characteristics according to Alternate Mediterranean Diet (aMed) score quintiles for participants in the Atherosclerosis Risk in Communities study.

	Quintiles of aMed score: aMed score range (*n* participants)				
Baseline characteristic	Quintile 1: 0–2 (*n* = 2,340)	Quintile 2: 3–4 (*n* = 4,573)	Quintile 3: 5 (*n* = 2,152)	Quintile 4: 6 (*n* = 1,589)	Quintile 5: 7–9 (*n* = 1,247)
Age (years)	53 ± 5.6	54 ± 5.7	54 ± 5.6	54 ± 5.8	54 ± 5.6
Female	1,365 (56%)	2,558 (56%)	1,226 (57%)	928 (58%)	678 (54%)
White	1,872 (77%)	3,407 (75%)	1,566 (73%)	1,206 (76%)	955 (76%)
Education					
Less than high school	637 (26%)	1,094 (24%)	417 (19%)	236 (15%)	163 (13%)
High school or equivalent	1,079 (43%)	1,888 (41%)	896 (42%)	619 (39%)	468 (38%)
More than high school	714 (29%)	1,591 (35%)	839 (39%)	734 (46%)	616 (49%)
Smoking status					
Current	774 (32%)	1,294 (28%)	495 (23%)	299 (19%)	237 (19%)
Former	700 (29%)	1,372 (30%)	700 (32%)	562 (36%)	446 (36%)
Never	955 (39%)	1,906 (42%)	957 (45%)	724 (46%)	564 (45%)
Body mass index (kg/m^2^)	27.5 ± 5.4	27.3 ± 5.1	27.6 ± 5.2	27.2 ± 5.0	26.7 ± 4.7
BMI categories					
Normal (18.5 to <25)	839 (35%)	1,563 (34%)	703 (33%)	560 (35%)	482 (39%)
Overweight (25 to <30)	940 (37%)	1,829 (40%)	875 (41%)	655 (41%)	491 (39%)
Obese (≥30)	632 (26%)	1,126 (25%)	558 (26%)	359 (23%)	262 (21%)
Fasting glucose (mmol/L)	5.5 ± 0.55	5.5 ± 0.52	5.5 ± 0.53	5.4 ± 0.52	5.4 ± 0.50
Hypertensive^a^	690 (29%)	1,413 (31%)	706 (33%)	479 (30%)	349 (28%)
LDL-cholesterol (mmol/L)	3.5 ± 0.99	3.5 ± 1.01	3.6 ± 1.04	3.5 ± 1.02	3.5 ± 0.99
Physical activity score^b^	2.3 ± 0.74	2.4 ± 0.77	2.5 ± 0.79	2.6 ± 0.81	2.7 ± 0.85

Results are presented as mean ± standard deviation for continuous variables and *n* (%) for categorical variables. Column totals may not add up to 100% due to rounding.

^a^Hypertension status determined if systolic blood pressure ≥140 mm Hg, diastolic blood pressure ≥90 mm Hg, or self-reported anti-hypertension medication usage^26^.

^b^Physical activity score (1-lowest to 5-highest) calculated based on intensity and time of leisure sport and exercise^24,25^.

Participants in higher quintiles of aMed scores had qualitatively higher intakes of total energy, percent of energy from carbohydrates, and percent of energy from protein compared to participants in lower aMed score quintiles (Table 2). Percent of energy from total and saturated fat was lower for higher quintiles of aMed scores, while percent of energy from monounsaturated and polyunsaturated fats were similar across quintiles. Fiber, sodium, potassium, and magnesium were higher for higher quintiles and cholesterol was lower at higher quintiles. The aMed components of fruits, vegetables, nuts, whole grains, legumes, and fish were higher at higher quintiles of aMed scores but MUFA:SFA and red and processed meats were similar. The number of alcoholic drinks consumed per week was higher at higher quantiles.

**Table 2:** Dietary intake according to Alternate Mediterranean Diet (aMed) score quintiles in the Atherosclerosis Risk in Communities study.

	Quintile of aMed Score: aMed score range (*n* participants)				
Nutrient	Quintile 1: 0–2 (*n* = 2,430)	Quintile 2: 3–4 (n = 4,573)	Quintile 3: 5 (*n* = 2,152)	Quintile 4: 6 (*n* = 1,589)	Quintile 5: 7–9 (*n* = 1,247)
Energy intake (kcal/day)	1,432 ± 520.0	1,566 ± 537.5	1,709 ± 553.0	1,803 ± 564.7	1,877 ± 521.8
Macronutrients					
%E^a^ carbohydrate	47 ± 9.0	49 ± 8.7	50 ± 7.9	51 ± 7.8	52 ± 7.1
%E protein	17 ± 3.8	18 ± 3.7	18 ± 3.7	19 ± 3.5	19 ± 3.3
%E fat	35 ± 6.3	33 ± 6.0	32 ± 5.8	31 ± 5.7	30 ± 5.4
%E saturated fat	13 ± 2.7	12 ± 2.5	11 ± 2.3	11 ± 2.3	10 ± 2.1
%E monounsaturated fat	13 ± 2.7	13 ± 2.7	12 ± 2.6	12 ± 2.6	12 ± 2.5
%E polyunsaturated fat	5 ± 1.2	5 ± 1.2	5 ± 1.2	5 ± 1.1	5 ± 1.1
Fiber (g/1000 kcal)	8 ± 2.7	10 ± 3.2	12 ± 3.4	13 ± 3.4	14 ± 3.7
Cholesterol (mg/1000 kcal)	163 ± 55.8	154 ± 52.4	151 ± 49.3	146 ± 44.0	138 ± 42.78
Micronutrients					
Sodium (mg/1000 kcal)	877 ± 185.2	905 ± 182.7	929 ± 171.5	951 ± 166.5	963 ± 162.4
Potassium (mg/1000 kcal)	1,510 ± 378.8	1,630 ± 378.4	1,724 ± 364.1	1,774 ± 336.0	1,815 ± 317.3
Magnesium (mg/1000 kcal)	142 ± 35.1	155 ± 34.6	166 ± 34.3	172 ± 32.6	180 ± 33.1
Calcium (mg/1000 kcal)	398 ± 177.1	401 ± 167.7	411 ± 151.1	418 ± 145.3	410 ± 135.4
aMed score components					
Vegetables (cups/day)	1.3 ± 0.81	2.1 ± 1.34	3.0 ± 1.76	3.6 ± 2.04	4.0 ± 2.49
Fruits (servings/day)	1.2 ± 0.88	1.8 ± 1.26	2.4 ± 1.45	2.8 ± 1.65	3.1 ± 1.38
Nuts (ounces/week)	1.1 ± 1.80	2.1 ± 2.89	2.9 ± 3.36	3.4 ± 3.40	4.4 ± 4.10
Whole grains (servings/day)	0.5 ± 0.55	0.8 ± 0.82	1.1 ± 0.92	1.3 ± 0.92	1.6 ± 0.94
Legumes (cups/day)	0.7 ± 0.50	1.0 ± 0.75	1.3 ± 0.88	1.5 ± 1.07	1.8 ± 1.12
Fish (servings/week)	1.0 ± 0.92	1.7 ± 1.48	2.5 ± 2.21	3.1 ± 2.92	3.7 ± 2.44
MUFA:SFA^b^	1.0 ± 0.31	1.1 ± 0.15	1.1 ± 0.15	1.1 ± 0.15	1.2 ± 0.15
Red and processed meat (servings/day)	1.1 ± 0.70	1.1 ± 0.69	1.1 ± 0.78	1.0 ± 0.85	0.9 ± 0.72
Alcohol (g/day)^c^	0 (0–4)	0 (0–6)	0 (0–6)	0 (0–7)	0 (0–10)
Drinks per week^d^	2.5 (0–7.5)	2.5 (0–7.0)	2.5 (0–7.0)	3.0 (0.5–7.0)	4.0 (1.0–8.0)

Results are presented as mean ± standard deviation, unless noted otherwise. Dietary intakes are self-reported means of visit 1 and visit 3 via self-reported via a food frequency questionnaire. Visit 1 dietary intake was used if incident diabetes or censoring occurred before visit 3. A detailed description of portion sizes is available in Supplementary Table S1.

^a^%E; percent of total energy.

^b^Monounsaturated to saturated fat ratio.

^c^Median (25th percentile–75th percentile) reported for alcohol in units of g/day. A standard drink contains about 14 g of pure alcohol.

^d^Median and (25th percentile–75th percentile) reported for servings (drinks of alcohol) per week.

**Fig. 1:**
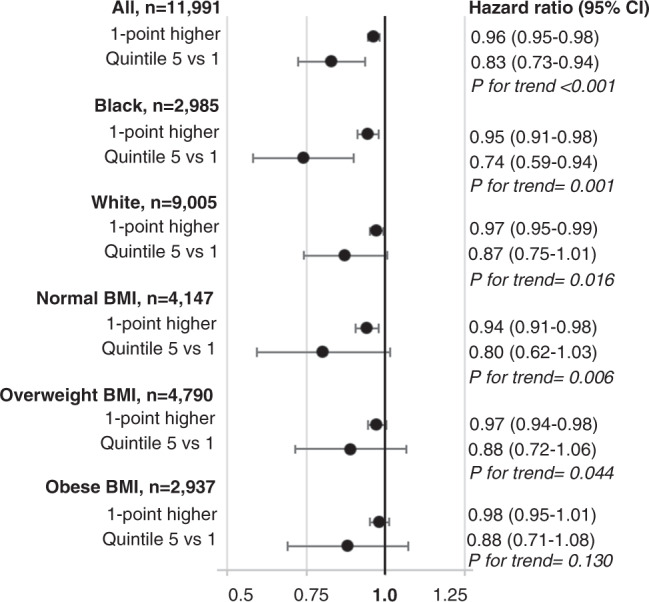
Adjusted hazard ratios for Alternate Mediterranean Diet (aMed) scores and incident diabetes for the overall population and according to race and BMI categories in the Atherosclerosis Risk in Communities study. Results are represented as hazard ratios and 95% confidence intervals from Cox regression models adjusted for total energy intake, age, sex, race and study center (center only for the race-specific analyses), and education level. The first point estimate within each subgroup represents the risk of incident diabetes per 1-point higher in aMed scores. The second point estimate within each subgroup represents the risk of incident diabetes for those in the fifth quintile vs the first quintile (reference group). *P*-values for trend were calculated from Wald tests modeling aMed quintiles as an ordinal variable.

**Fig. 2:**
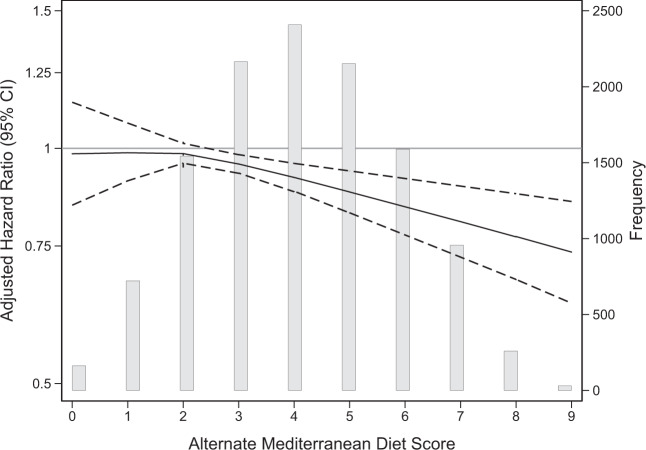
Alternate Mediterranean Diet (aMed) score distribution and adjusted hazard ratios for incident diabetes in the Atherosclerosis Risk in Communities study. The solid line indicates the hazard ratio for incident diabetes estimated via a Cox regression model with Alternate Mediterranean Diet scores modeled continuously as a linear spline with a knot at a score of 2 points, adjusted for total energy intake, age, sex, race and study center, and education level. The dashed lines indicate the 95% confidence interval. The grey bars represent the number of participants with each alternate Mediterranean diet score.

During a median of follow-up of 22 years, there were 4,024 incident cases of diabetes among the 11,991 participants. The overall incidence rate during follow-up was 1.7 diabetes cases per 100 person-years. Higher quintiles of aMed scores were associated with lower incident diabetes risk (trend *p* < 0.001) after controlling for energy intake and socio-demographic factors [Model 1 HR (95% CI) for quintile 5 vs 1: 0.83 (0.73–0.94); Fig. 1; Supplementary Table S2). The HR for quintile 5 vs. 1 was attenuated after adjusting for physical activity and smoking [Model 2 HR (95% CI): 0.88 (0.77–0.99)]. After further adjusting for clinical measures, there was no direct association between aMed score quintiles and diabetes [Model 3 HR (95% CI): 0.94 (0.82–1.07)]. Trends across quintiles remained statistically significant for Models 2 and 3 (*p* = 0.005 and *p* = 0.03, respectively; Supplementary Table S2). A 1-point higher aMed score was associated with a lower risk of incident diabetes after adjusting for energy intake and socio-demographic factors [Model 1 HR (95% CI): 0.96 (0.95–0.98); Fig. 1], behavioral risk factors [Model 2 HR (95% CI): 0.97 (0.96–0.99], and clinical measures [Model 3 HR (95% CI): 0.98 (0.96–0.99); Supplementary Table S2]. There was a linear inverse relationship between aMed scores and incident diabetes risk for aMed scores at and above a score of 2-points (*p* < 0.001; Fig. 2).

There were stronger inverse associations between aMed scores and diabetes risk for black compared to white participants (Fig. 2; Supplementary Table S3; *p* value for interaction <0.001). Associations were significant for participants who had a normal BMI, attenuated for participants who were overweight, and not significant for participants who were obese (Fig. 1; Supplementary Table S4; interaction *p* value < 0.01). The relationship between aMed scores and incident diabetes did not differ by sex or education level (interaction *p* values > 0.05).

Higher component scores for nuts [Model 1 HR (95% CI): 0.92 (0.86–0.98] and legumes [Model 1 HR (95% CI): 0.92 (0.86–0.98)], representing intake above sex-specific medians, and higher component scores for alcohol [Model 1 HR (95% CI): 0.83 (0.75–0.91)], representing moderate intake, were associated with a lower risk of incident diabetes after adjusting for total energy intake and socio-demographic factors. Higher component scores for red and processed meat (Model 1 HR (95% CI): 0.91 (0.84–0.97)], indicating intake below the sex-specific median, was also associated with lower risk of incident diabetes. Component scores for whole grains, vegetables, fruits, MUFA:SFA, and fish were not independently associated with diabetes risk. Results were similar for Model 2 but only alcohol remained significant in Model 3 [HR (95% CI): 0.81 (0.74–0.90)]. Results were similar when all aMed components were included in the models.

## Discussion

Higher Mediterranean-style eating pattern scores were inversely associated with risk of incident diabetes in this prospective cohort study of 11,991 middle-aged U.S. adults followed for a median of 22 years. The results were attenuated but persisted after additional adjustment for health behaviors and clinical factors related to diabetes risk. Associations between aMed scores and incident diabetes were stronger among black compared to white participants, were stronger for normal weight compared to overweight or obese participants, and were not different for males and females. Higher nut and legume intake, moderate alcohol intake, and lower red and processed meat intake were the main drivers of the noted associations.

Associations between Mediterranean pattern scores and diabetes risk in U.S. cohorts are inconsistent. We found that Mediterranean pattern scores are associated with a lower risk of diabetes in a U.S. population of blacks and whites by up to 17%. Diabetes risk reductions (up to 25%) associated with Mediterranean pattern scores were previously noted in a cohort of mostly white high socioeconomic U.S. men (Health Professionals Follow-up Study; *n* = 41,615)^29^. In a smaller but more ethnically diverse U.S. cohort, the Multi-Ethnic Study of Atherosclerosis (*n* = 5,290), there was no association between Mediterranean pattern scores and diabetes risk in the overall population or within racial subgroups^30^. The larger sample size of the ARIC study allowed for more precise white vs black comparisons than these prior studies. We found stronger associations between Mediterranean pattern adherence and incident diabetes for black compared to white participants. This is contrary to previous research which showed inverse associations between a Dietary Approaches to Stop Hypertension-style eating pattern and diabetes risk only for white individuals in a cohort that was comprised largely of black and Hispanic individuals^31^. There is a need for follow-up research regarding the finding that black individuals may particularly benefit from adherence to a Mediterranean pattern because of the high risk of diabetes in this racial subgroup^27^.

Unlike data from U.S. cohorts, associations between higher Mediterranean pattern scores and reduced diabetes risk are consistent from Mediterranean/European cohorts^14–16^. One hypothesis is that this may be due to cultural practices of a Mediterranean lifestyle. Lifestyle and food choices have changed in the Mediterranean region since Ancel Key’s discovery of the cardioprotective Mediterranean pattern^12^. Modernized farming practices and industrialization of the food supply have led to increased BMI and higher cardiovascular disease risk in the same geographic regions that Keys first mapped out^32^. However, daily physical activity, close social networks, shared family meals, adequate rest, and abundant social exchanges of a Mediterranean lifestyle still persist as protective disease factors into the twenty-first century^13^. Aside from physical activity, these factors were not measured in ARIC so it is difficult to determine whether these behaviors would modify associations between Mediterranean pattern adherence and chronic disease risk in our population.

Obtaining and maintaining a healthy body weight is recognized as the most influential modifiable risk factor for type 2 diabetes prevention^17^. We found that associations between aMed score and incident diabetes was stronger among participants with a normal BMI at baseline. The results of this analysis suggest that the detrimental health implications of being overweight or obese override potential health benefits of a Mediterranean pattern. This theme is previously noted in the ARIC study^33,34^, as well as other U.S. populations^27^, for associations between healthy eating patterns and various chronic disease outcomes. Additionally, previous controlled feeding trials in individuals without diabetes showed little improvement in fasting markers of glycemic control when adopting a weight-maintenance Mediterranean pattern^7^ or other heart healthy eating pattern^33–37^ in the absence of body weight reductions >2 kilograms. However, markers of glycemic control can improve regardless of eating pattern composition in trials that prescribe intentional weight loss for participants^38,39^. Therefore, our results align with those of randomized controlled trials which suggest that adopting a healthy eating pattern in the absence of weight loss may not meaningfully reduce diabetes risk or associated risk factors in overweight or obese populations. While adherence to a healthy eating pattern high in fruits, vegetables, and whole grains could potentially result in lower diabetes risk among those with normal BMI, discussion of calorie restriction to achieve and maintain a healthy body weight should remain at the forefront of diabetes prevention.

The ARIC FFQ was not a priori designed to assess adherence to a Mediterranean pattern. The FFQ contains no questions about olive oil intake, which is the main staple of a Mediterranean pattern. Further, limited variation in fruit, vegetable, and whole-grain eating habits of U.S. populations, as well as the lack of adequate whole-grain related FFQ questions, may not be sufficient to correctly identify independent associations with diabetes. Due to the aMed rank-based scoring system used to assess Mediterranean pattern adherence, no quintile met commonly recommended food group intake thresholds of a more traditional Mediterranean patterns for nuts, fats, vegetables, or whole grains^1,3^. Further, a traditional Mediterranean pattern is commonly recognized to be high in total and/or monounsaturated fat (up to 40% and 20% of total energy, respectively)^3,4^. The higher quintiles in this study reported lower total fat and saturated fat intakes than the lower quintiles with limited variability in mono- and polyunsaturated fat across quintiles. To note, about half of monounsaturated fatty acids consumed by U.S. populations come from red meat, not olive oil. The associations noted between aMed scores and diabetes risk in this study are not applicable to a traditional high total and monounsaturated fat (from olive oil) Mediterranean pattern. Switching to a high-fat eating pattern of any kind could potentially cause weight-gain and increase diabetes risk because all fat types are energy dense^40^.

Our study can address previously noted gaps in the literature about eating patterns and chronic disease risk. The observed inverse associations between Mediterranean pattern adherence and incident diabetes may be more precise and generalizable to the U.S. population than previous studies because the ARIC study is a large cohort of both men and women, and blacks and whites, representing four different U.S. regions. We also used a highly sensitive measure of incident diabetes (doctor diagnosis, medication usage, and blood glucose concentrations), although largely self-reported^23^. Although type 1 vs type 2 diabetes cases were indistinguishable via these ascertainment methods, 90–95% of diabetes cases in the U.S. are type 2 diabetes^41^. Further, type 2 diabetes most often presents in individuals over the age of 45^41^. We excluded diabetes cases at baseline and participant age at enrollment was >45 years. Therefore, it is assumed that incident diabetes cases in this sample are largely type 2 diabetes. Further, while dietary intake assessments were self-reported, using the average of two measurements (visit 1 and visit 3) is expected to reduce measurement error and increase precision of exposure-outcome associations^42^. The temporal nature of assessing Mediterranean pattern adherence at baseline with a median of 22 follow-up years is another strength of this study.

Unmeasured and residual confounding in observational studies can remain after multivariable adjustments. Specifically, associations between Mediterranean pattern adherence and diabetes were negated in participants who were overweight or obese. However, energy intake was highest in the fifth quintile with limited variation in BMI and physical activity across quintiles. Energy calculations from food frequency questionnaires tend to be unreliable and there was no objective measure of energy intake (such as doubly labeled water) used in the ARIC study to validate the energy intake reported in Table 2. To address this source of potential confounding, the multivariable regression models were adjusted for total energy intake. However, more generally, individuals in the U.S. who adhere to a healthy eating pattern often have other healthy lifestyle behaviors such as being physically active, refraining from smoking, and have financial and educational means to make healthier lifestyle choices. This extent of residual confounding after adjusting for these factors is unknown.

An eating pattern high in fruits, vegetables, whole grains, legumes, nuts, and fish, and moderate in alcohol, similar to the Mediterranean-style eating pattern assessed in this analysis and recommended by the 2015–2020 Dietary Guidelines for Americans, was associated with an overall lower risk of diabetes in a community-based U.S. population. This association was particularly strong for black and normal weight individuals but was absent for individuals who were overweight or obese. Increased awareness and promotion of healthy eating patterns for diabetes prevention in predominantly black communities may reduce disease burden. Future research is needed to assess if a calorically restrictive Mediterranean-style eating pattern, resulting in clinically meaningful weight loss, can reduce future diabetes risk in individuals who are overweight or obese. Adhering to a healthy eating pattern is an important component of an overall healthy and active lifestyle to obtain and maintain a healthy body weight and to reduce risk of adult-onset diabetes for the U.S. population.

## Supplementary information

Supplemental Table S1-S4, Supplemental Figure S1

## Data Availability

Data are available upon request from the National Heart, Lung, and Blood Institute Biologic Specimens and Data Repository Information Coordinating Center (BioLINCC) (accession number: HLB00020019b).

## Code availability

Statistical code is available upon request from the corresponding author.
