# Supplementary material for: Adherence to a Mediterranean-style eating pattern and risk of diabetes in a U.S. prospective cohort study
Source: Nutr Diabetes. 2020 Mar 20;10:8. doi: 10.1038/s41387-020-0113-x (PMC7083875; doi:10.1038/s41387-020-0113-x)
Supplement: Supplementary file 1 — Supplemental Table S1-S4, Supplemental Figure S1 [file 41387_2020_113_MOESM1_ESM.docx]

**Supplementary Table S1**: Allocation of food frequency questionnaire items for Alternate Mediterranean Diet (aMed) score from the Atherosclerosis Risk in Communities (ARIC) study

| **Scoring category** | **1 point if…** | **Food and beverage items from ARIC food frequency questionnaire** |
| --- | --- | --- |
| Vegetables | above sex-specific median  (F= 1.16 c./day,  M= 0.95 c./day) | Broccoli; 1/2 c.*  Cabbage, cauliflower, brussels sprouts; 1/2 c.  Carrots; 1 whole or 1/2 c. cooked  Corn; 1 ear or 1/2 c.  Spinach, collards, or other greens, but do not include lettuce; 1/2 c.  Dark yellow, winter squash such as acorn, butternut; 1/2 c.  Tomatoes; 1, or tomato juice; 4 oz.^†^  Sweet potatoes; 1/2 c. |
| Fruit | above sex-specific median  (F= 2.00 servings/day,  M= 1.59 servings/day) | Fresh apples or pears; 1  Oranges; 1  Orange or grapefruit juice; small glass  Peaches, apricots, plums; 1 fresh or 1/2 c. canned or dried  Bananas; 1  Other fruits; 1 fresh or 1/2 c. canned, including fruit cocktail |
| Whole grain | above sex-specific median  (F= 0.75 servings/day,  M= 0.64 servings/day) | Cooked cereals such as oatmeal, grits, cream of wheat; 1/2 c.  Dark or whole grain bread; 1 slice |
| Nuts | above sex-specific median  (F= 0.17 oz./day,  M= 0.25 oz./day) | Peanut butter; 1 tbsp^‡^  Nuts; 1 oz. |
| Legumes | above sex-specific median  (F= 0.46 c./day,  M= 0.50 c./day) | String or green beans; 1/2 c.  Peas or lima beans; 1/2 c. fresh, frozen or canned  Baked beans or lentils, dried cooked or canned, such as pinto, blackeye, baked beans; 1/2 c. |
| Red and processed meat | below sex-specific median  (F= 0.78 servings/day,  M= 1.14 servings/day) | Hamburgers; 1  Hot Dogs; 1  Processed meats: sausage, salami, bologna, etc.; piece or slice  Bacon; 2 slices  Beef, pork, or lamb as a sandwich or mixed dish, stew, casserole, lasagna, or in spaghetti sauce, etc.  Beef, pork, or lamb as a main dish, steak, roast, ham, etc. |
| Fish | above sex-specific median  (F= 0.24 servings/day,  M= 0.21 servings/day) | Canned tuna fish; 3-4 oz.  Dark meat fish, such as salmon, mackerel, swordfish, sardines, bluefish; 3-5 oz.  Other fish, such as cod, perch, catfish, etc.; 3-5 oz.  Shrimp, lobster, scallops as a main dish |
| Alcohol | F: 5-15 g/day  M: 10-25 g/day | Calculated from nutrient output |
| MUFA:SFA | above sex-specific median  (F= 1.07,  M= 1.10) | Calculated from nutrient output |
| TOTAL | 9 points |  |

* Cup

^†^ Ounce

^‡^ Tablespoon **Supplementary Table S2**: Hazard ratios for Alternate Mediterranean Diet (aMed) scores and incident diabetes in the Atherosclerosis Risk in Communities study

|  | **Quintile 1** | **Quintile 2** | **Quintile 3** | **Quintile 4** | **Quintile 5** | **P-trend** | **1-point higher** |
| --- | --- | --- | --- | --- | --- | --- | --- |
| **Model 1** |  |  |  |  |  |  |  |
| Total (n) | 2,430 | 4,573 | 2,152 | 1,589 | 1,247 |  | 11,991 |
| Cases (n) | 843 | 1,562 | 717 | 506 | 396 |  | 4,024 |
| Hazard ratio  (95% CI) | REF | 0.97  (0.89-1.06) | 0.90  (0.82-1.00) | 0.84  (0.75-0.94) | 0.83  (0.73-0.94) | <0.001 | 0.96  (0.95-0.98) |
| Incidence rate* | 1.8 | 1.8 | 1.7 | 1.6 | 1.6 |  | 1.7 |
| **Model 2** |  |  |  |  |  |  |  |
| Total (n) | 2,425 | 4,554 | 2,149 | 1,584 | 1,244 |  | 11,956 |
| Cases (n) | 841 | 1557 | 716 | 504 | 395 |  | 4,013 |
| Hazard ratio  (95% CI) | REF | 0.99  (0.91-1.08) | 0.93  (0.84-1.03) | 0.88  (0.79-0.99) | 0.88  (0.77-0.99) | 0.005 | 0.97  (0.96-0.99) |
| Incidence rate* | 1.8 | 1.8 | 1.7 | 1.6 | 1.6 |  | 1.7 |
| **Model 3** |  |  |  |  |  |  |  |
| Total (n) | 2,324 | 4,360 | 2,050 | 1,521 | 1,196 |  | 11,451 |
| Cases (n) | 796 | 1479 | 672 | 481 | 376 |  | 3,804 |
| Hazard ratio  (95% CI) | REF | 1.02  (0.94-1.11) | 0.89  (0.80-0.99) | 0.91  (0.81-1.02) | 0.94  (0.82-1.07) | 0.03 | 0.98  (0.96-0.99) |
| Incidence rate* | 1.8 | 1.8 | 1.7 | 1.5 | 1.6 |  | 1.7 |

Results are presented as hazard ratios and 95% confidence intervals estimated from Cox proportional hazards regression models. Model 1 included energy intake, age, sex, race-center, and education level as covariates. Model 2 further included smoking and physical activity and Model 3 further included clinical measures including fasting glucose, hypertension status (yes/no), low-density lipoprotein cholesterol, BMI category, and family history of diabetes (yes/no). P-values for trend were calculated by modeling aMed quintile as an ordinal variable.

* Incidence rate of diabetes per 100 person-years

**Supplementary Table S3**: Hazard ratios for Alternate Mediterranean Diet (aMed) scores and incident diabetes in the Atherosclerosis Risk in Communities study stratified by race

|  | **Quintile 1** | **Quintile 2** | **Quintile 3** | **Quintile 4** | **Quintile 5** | **P-trend** | **1-point higher** |
| --- | --- | --- | --- | --- | --- | --- | --- |
| **Black** |  |  |  |  |  |  |  |
| **Model 1** |  |  |  |  |  |  |  |
| Total (n) | 558 | 1,116 | 586 | 383 | 292 |  | 2,985 |
| Cases (n) | 247 | 485 | 248 | 148 | 116 |  | 1,244 |
| Incidence rate* | 2.7 | 2.5 | 2.4 | 2.1 | 2.1 |  | 2.4 |
| Hazard ratio  (95% CI) | REF | 0.90  (0.77-1.05) | 0.86  (0.71-1.03) | 0.73  (0.59-0.90) | 0.74  (0.59-0.94) | 0.001 | 0.95  (0.91-0.98) |
| **Model 2** |  |  |  |  |  |  |  |
| Total (n) | 557 | 1,163 | 585 | 381 | 292 |  | 2,978 |
| Cases (n) | 247 | 483 | 247 | 147 | 116 |  | 1,240 |
| Incidence rate | 2.7 | 2.5 | 2.4 | 2.1 | 2.1 |  | 2.4 |
| Hazard ratio  (95% CI) | REF | 0.90  (0.77-1.05) | 0.86  (0.71-1.03) | 0.73  (0.59-0.91) | 0.75  (0.59-0.94) | 0.002 | 0.95  (0.91-0.98) |
| **Model 3** |  |  |  |  |  |  |  |
| Total (n) | 494 | 1,052 | 534 | 349 | 272 |  | 2,701 |
| Cases (n) | 221 | 439 | 220 | 135 | 107 |  | 1,122 |
| Incidence rate | 2.7 | 2.5 | 2.3 | 2.1 | 2.1 |  | 2.4 |
| Hazard ratio  (95% CI) | REF | 0.92  (0.78-1.08) | 0.85  (0.70-1.04) | 0.76  (0.61-0.96) | 0.81  (0.63-1.03) | 0.018 | 0.96  (0.92-0.99) |
| **White** |  |  |  |  |  |  |  |
| **Model 1** |  |  |  |  |  |  |  |
| Total (n) | 1,872 | 3,407 | 1,566 | 1,206 | 955 |  | 9,006 |
| Cases (n) | 569 | 1077 | 469 | 358 | 280 |  | 2,780 |
| Incidence rate | 1.6 | 1.6 | 1.5 | 1.4 | 1.4 |  | 1.5 |
| Hazard ratio  (95% CI) | REF | 0.99  (0.90-1.10) | 0.92  (0.81-1.04) | 0.90  (0.79-1.03) | 0.87  (0.75-1.01) | 0.016 | 0.97  (0.95-0.99) |
| **Model 2** |  |  |  |  |  |  |  |
| Total (n) | 1,868 | 3,391 | 1,564 | 1,203 | 952 |  | 8,978 |
| Cases (n) | 594 | 1,074 | 469 | 357 | 279 |  | 2,773 |
| Incidence rate | 1.6 | 1.6 | 1.5 | 1.4 | 1.4 |  | 1.5 |
| Hazard ratio  (95% CI) | REF | 1.02  (0.92-1.13) | 0.97  (0.85-1.09) | 0.96  (0.83-1.10) | 0.94  (0.81-1.10) | 0.23 | 0.99  (0.96-1.00) |
| **Model 3** |  |  |  |  |  |  |  |
| Total (n) | 1,831 | 3,311 | 1,517 | 1,174 | 924 |  | 8,757 |
| Cases (n) | 575 | 1,040 | 453 | 346 | 269 |  | 2,683 |
| Incidence rate | 1.6 | 1.6 | 1.5 | 1.4 | 1.4 |  | 1.5 |
| Hazard ratio  (95% CI) | REF | 1.05  (0.95-1.17) | 0.88  (0.77-0.99) | 0.98  (0.85-1.12) | 0.98  (0.84-1.12) | 0.24 | 0.98  (0.96-1.00) |

Results are presented as hazard ratios and 95% confidence intervals estimated from Cox proportional hazards regression models. Model 1 included energy intake, age, sex, race-center, and education level as covariates. Model 2 further included smoking and physical activity and Model 3 further included clinical measures including fasting glucose, hypertension status (yes/no), low-density lipoprotein cholesterol, BMI category, and family history of diabetes (yes/no). P-values for trend were calculated by modeling aMed quintile as an ordinal variable. Likelihood ratio test for interaction p<0.001.

* Incidence rate of diabetes per 100 person-years

**Supplementary Table S4**: Hazard ratios for Alternate Mediterranean Diet (aMed) scores and incident diabetes in the Atherosclerosis Risk in Communities study stratified by body mass index category

|  | **Quintile 1** | **Quintile 2** | **Quintile 3** | **Quintile 4** | **Quintile 5** | **P-trend** | **1-point higher** |
| --- | --- | --- | --- | --- | --- | --- | --- |
| **Normal**  **18.5-<25 kg/m^2^** |  |  |  |  |  |  |  |
| **Model 1** |  |  |  |  |  |  |  |
| Total (n) | 839 | 1,563 | 703 | 560 | 482 |  | 4,147 |
| Cases (n) | 194 | 342 | 140 | 100 | 95 |  | 871 |
| Incidence rate* | 1.1 | 1.0 | 0.9 | 0.8 | 0.9 |  | 1.0 |
| Hazard ratio  (95% CI) | REF | 0.91  (0.76-1.09) | 0.81  (0.65-1.02) | 0.69  (0.54-0.89) | 0.80  (0.62-1.03) | 0.006 | 0.94  (0.91-0.98) |
| **Model 2** |  |  |  |  |  |  |  |
| Total (n) | 838 | 1,557 | 701 | 559 | 481 |  | 4,136 |
| Cases (n) | 193 | 341 | 139 | 100 | 95 |  | 868 |
| Incidence rate | 1.1 | 1.0 | 0.9 | 0.8 | 0.9 |  | 1.0 |
| Hazard ratio  (95% CI) | REF | 0.94  (0.78-1.12) | 0.86  (0.69-1.08) | 0.75  (0.58-0.96) | 0.88  (0.67-1.14) | 0.06 | 0.96  (0.92-0.99) |
| **Model 3** |  |  |  |  |  |  |  |
| Total (n) | 814 | 1,504 | 681 | 543 | 465 |  | 4,007 |
| Cases (n) | 186 | 321 | 134 | 99 | 92 |  | 832 |
| Incidence rate | 1.1 | 1.0 | 0.9 | 0.8 | 0.9 |  | 1.0 |
| Hazard ratio  (95% CI) | REF | 0.89  (0.74-1.07) | 0.85  (0.67-1.06) | 0.72  (0.56-0.93) | 0.94  (0.72-1.23) | 0.15 | 0.96  (0.93-1.00) |
| **Overweight**  **25-<30** **kg/m^2^** |  |  |  |  |  |  |  |
| **Model 1** |  |  |  |  |  |  |  |
| Total (n) | 940 | 1,829 | 875 | 655 | 491 |  | 4,790 |
| Cases (n) | 330 | 652 | 319 | 221 | 169 |  | 1,691 |
| Incidence rate | 1.9 | 1.9 | 1.9 | 1.6 | 1.7 |  | 1.8 |
| Hazard ratio  (95% CI) | REF | 1.01  (0.89-1.16) | 0.98  (0.84-1.15) | 0.87  (0.73-1.04) | 0.88  (0.72-1.06) | 0.04 | 0.97  (0.94-0.98) |
| **Model 2** |  |  |  |  |  |  |  |
| Total (n) | 937 | 1,824 | 874 | 652 | 489 |  | 4,776 |
| Cases (n) | 329 | 650 | 319 | 219 | 168 |  | 1,685 |
| Incidence rate | 1.9 | 1.9 | 1.9 | 1.6 | 1.7 |  | 1.8 |
| Hazard ratio  (95% CI) | REF | 1.03  (0.90-1.18) | 1.02  (0.87-1.20) | 0.91  (0.76-1.09) | 0.93  (0.77-1.13) | 0.20 | 0.98  (0.95-1.01) |
| **Model 3** |  |  |  |  |  |  |  |
| Total (n) | 892 | 1,750 | 835 | 626 | 471 |  | 4,574 |
| Cases (n) | 307 | 619 | 302 | 206 | 162 |  | 1,596 |
| Incidence rate | 1.9 | 1.9 | 1.9 | 1.6 | 1.7 |  | 1.8 |
| Hazard ratio  (95% CI) | REF | 1.06  (0.92-1.22) | 0.97  (0.83-1.15) | 0.98  (0.81-1.17) | 1.01  (0.82-1.23) | 0.62 | 0.99 (0.96-1.02) |
| **Obese**  **≥30** **kg/m^2^** |  |  |  |  |  |  |  |
| **Model 1** |  |  |  |  |  |  |  |
| Total (n) | 632 | 1,126 | 558 | 359 | 262 |  | 2,937 |
| Cases (n) | 316 | 562 | 257 | 184 | 131 |  | 1,450 |
| Incidence rate | 3.1 | 3.1 | 2.7 | 3.1 | 2.9 |  | 3.0 |
| Hazard ratio  (95% CI) | REF | 1.00  (0.87-1.15) | 0.85  (0.72-1.00) | 0.97  (0.80-1.17) | 0.88  (0.71-1.08) | 0.13 | 0.98  (0.95-1.01) |
| **Model 2** |  |  |  |  |  |  |  |
| Total (n) | 631 | 1,119 | 558 | 358 | 262 |  | 2,928 |
| Cases (n) | 316 | 560 | 257 | 184 | 131 |  | 1,448 |
| Incidence rate | 3.1 | 3.1 | 2.7 | 3.1 | 2.9 |  | 3.0 |
| Hazard ratio  (95% CI) | REF | 1.00  (0.87-1.15) | 0.84  (0.71-1.00) | 0.97  (0.80-1.17) | 0.87  (0.70-1.08) | 0.12 | 0.98  (0.95-1.01) |
| **Model 3** |  |  |  |  |  |  |  |
| Total (n) | 601 | 1,060 | 520 | 339 | 248 |  | 2,768 |
| Cases (n) | 300 | 533 | 236 | 175 | 121 |  | 1,365 |
| Incidence rate | 3.1 | 3.1 | 2.7 | 3.1 | 2.9 |  | 3.0 |
| Hazard ratio  (95% CI) | REF | 1.06  (0.92-1.22) | 0.88  (0.74-1.05) | 0.96  (0.79-1.17) | 0.87  (0.69-1.08) | 0.08 | 0.98  (0.95-1.01) |

Results are presented as hazard ratios and 95% confidence intervals estimated from Cox proportional hazards regression models. Model 1 included energy intake, age, sex, race-center, and education level as covariates. Model 2 further included smoking and physical activity and Model 3 further included clinical measures including fasting glucose, hypertension status (yes/no), low-density lipoprotein cholesterol, BMI category, and family history of diabetes (yes/no). P-values for trend were calculated by modeling aMed quintile as an ordinal variable. Likelihood ratio test for interaction p<0.01. Individuals in our final sample of 11,991 who were underweight were excluded from these analyses (n=117).

*Incidence rate of diabetes per 100 person-years

**Supplemental Figure S1**: Participant flow diagram

See powerpoint for figure

Participants who attended visit 1

n=15,792

Participants free of chronic disease at baseline

n=12,747

Participants with usable dietary intake data

n=12,204

Participants included in analysis

n=11, 991

Prevalent diabetes n=1,870

Prevalent coronary heart disease or previous myocardial infarction n=574

History of stroke n=152

History of cancer n=722

Asian American n=28

American Indian n=14

Blacks from Washington Co. n=25

Blacks from Minnesota n=19

Missing education level n=20

Missing follow-up time n=44

Missing food components of Alternate Mediterranean Diet Score n=63

Participants with implausible energy intake (<600 or >4200 kcal for men and <500 or >3600 for women) or ≥10 missing food items

n=270
